# Maternal and paternal tuberculosis is associated with increased asthma and respiratory symptoms in their offspring: a study from Northern Europe

**DOI:** 10.3389/falgy.2023.1193141

**Published:** 2023-06-08

**Authors:** Sanjay Gyawali, Juan Pablo López-Cervantes, Ane Johannessen, Thorarinn Gislason, Mathias Holm, Christer Janson, Rain Jögi, Lars Modig, Vivi Schlünssen, Tehmina Mustafa, Cecilie Svanes

**Affiliations:** ^1^ Department of Global Public Health and Primary Care, Centre for International Health, University of Bergen, Bergen, Norway; ^2^ Department of Occupational Medicine, Haukeland University Hospital, Bergen, Norway; ^3^ Faculty of Medical, University of Iceland, Reykjavik, Iceland; ^4^ Department of Sleep, Landspitali University Hospital, Reykjavik, Iceland; ^5^ Occupational and Environmental Medicine, School of Public Health and Community Medicine, Institute of Medicine, Sahlgrenska Academy, University of Gothenburg, Gothenburg, Sweden; ^6^ Department of Medical Sciences, Respiratory, Allergy and Sleep Research, Uppsala University, Uppsala, Sweden; ^7^ Lung Clinic, Tartu University Hospital, Tartu, Estonia; ^8^ Department of Public Health and Clinical Medicine, Sustainable Health, Umeå University, Umeå, Sweden; ^9^ Department of Public Health, Research Unit for Environment, Occupation and Health, Danish Ramazzini Centre, Aarhus University, Aarhus, Denmark; ^10^ Department of Thoracic Medicine, Haukeland University Hospital, Bergen, Norway

**Keywords:** asthma, tuberculosis, epigenetic, offspring asthma, generational study

## Abstract

**Background:**

Given the profound impact of tuberculosis (TB) on immunity and given murine studies suggesting that infections may influence immunity across generations, we hypothesize that parental TB might impact health and disease in future offspring.

**Objective:**

This study investigated the impact of maternal and paternal TB on offspring asthma and respiratory symptoms.

**Methods:**

We included data from the third follow-up of the Respiratory Health in Northern Europe study (RHINE). Information on own asthma status, asthma-like symptoms and other respiratory symptoms, as well as information about parental TB and asthma, were collected using standardized questionnaires. The associations between parental TB and RHINE participants' asthma and respiratory symptoms were analyzed using multiple logistic regression, with adjustment for parental education, smoking habits and asthma.

**Results:**

Of 8,323 study participants, 227 (2.7%) reported only paternal TB, 282 (3.4%) only maternal TB, and 33 (0.4%) reported that both parents had TB. We found a higher risk of asthma (aOR: 1.29, 95% CI: 1.05–1.57) in offspring with a history of parental TB as compared to offspring without parental TB., Parental TB was significantly associated with allergic asthma in offspring (aOR: 1.58, 95% CI: 1.29–2.05), while no significant association between parental TB and asthma without allergy (aOR: 1.00, 95% CI: 0.76–1.32) in offspring was observed.

**Conclusion:**

Results from this study indicate that parental TB might be a risk factor for offspring's asthma and respiratory symptoms. We raise the hypothesis that the immunological impact of infections might be transmitted to influence offspring phenotype in humans.

## 1. Introduction

Tuberculosis (TB) is caused mainly by *Mycobacterium tuberculosis* (Mtb), and represents a major global health threat, especially in low and middle-income countries (1). Asthma is a chronic inflammatory disease of the airways, which is associated with airway hyperresponsiveness leading to common symptoms of wheezing, breathlessness, chest tightness, and coughing (2). Pulmonary TB may cause significant lung damage and airflow obstruction by modifying the lung parenchyma and scaring of the lungs (3). This lung damage may increase the risk of developing asthma and other respiratory diseases in the affected person following TB (4–6).

Increasing evidence from murine studies shows that several infections in the parent generation can cause epigenetic modifications that may be passed on to succeeding generations (7–9). Tuberculosis is an infection that induces major immunological responses, and it seems plausible that this could contribute to train immunity also beyond the infected individual. At present, the association between parental TB and the risk of respiratory diseases in human offspring is unclear. To the best of our knowledge, there is only one previous human study by López-Cervantes et al. that is based on Norwegian health registries that have investigated the association of parental TB with offspring asthma. This study found that persons with parents with TB in childhood or young adulthood had more asthma as compared to persons with parental TB that occurred after these offspring were born (10). However, this study was limited to the offspring of persons with a history of TB, while there is a need to study the association of parental TB with offspring asthma in a general population. Given the profound impact of tuberculosis on immunity, we hypothesize that parental TB might impact respiratory health in their offspring.

In the current study, we investigated the association between parental TB and the prevalence of asthma and respiratory symptoms in their offspring in a Nordic-Baltic multicenter population-based study.

## 2. Methodology

### 2.1. Study design, settings, and participants

This analysis is based on data from standardized questionnaires administered in the Respiratory Health in Northern Europe study (RHINE, https://rhine.w.uib.no). The RHINE study is a follow-up study of the persons who participated in a postal survey of the European Community Respiratory Health Survey (ECRHS, www.ecrhs.org) in the early 1990’ies, from seven study centers in the Nordic-Baltic countries: Bergen, Aarhus, Reykjavik, Umeå, Uppsala, Gothenburg and Tartu. In ECRHS I, young adults aged 20–44 years were randomly selected from the population registries in the respective centers. Study participants were followed up after 10, 20, and 30 years, around the years 2000, 2010, and 2020/2022, respectively. Participants provided information on demographic characteristics, environmental factors, and respiratory symptoms in all study waves.

Questions regarding TB and respiratory diseases in their parents were included in the most recent RHINE follow-up (RHINE IV). Of the 8,965 participants from the seven centers, a total of 8,323 participants provided information about maternal and paternal TB and were included in the analyses.

Written consent was obtained from all the participants at each stage of the RHINE study, and the local ethical committee approved each study stage at each study center in accordance with national legislation.

### 2.2. Definition of exposure and outcomes

#### 2.2.1. Exposure

During RHINE IV, participants were asked whether their parents were ever treated for tuberculosis. Based on the question, a new variable was created and classified as follows:

1. Mother with TB: if participants answered “yes” to the mother having TB and “no” or “don’t know” to the father having TB.
2. Father with TB: if participants answered “yes” to the father having TB and “no” or “don’t know” to the mother having TB.
3. Both parents with TB: if participants answered “yes” to mother and father having TB.
4. Neither of the parents with TB: if participants answered “no” or “don’t know” to both mother and father having TB.

“Parental TB” includes 1, 2, and 3 of the above points.

#### 2.2.2. Outcomes

We define asthma (termed offspring asthma), in three different ways, according to Pekkanen and Sunyer (14, 15). Current asthma was defined as a positive response to the questions “Have you had an attack of asthma in the last 12 months?” and/or “Are you currently taking any medicine (including inhalers, aerosols, or tablets) for asthma?”. Asthma symptoms were defined based on affirmative answers to five self-reported symptoms common in asthmatics: wheezing, awoken with tightness in the chest, awoken with shortness of breath, awoken with an attack of cough, and breathlessness (Supplementary Table S1), all during the last 12 months. We assigned one point to each affirmative answer and categorized the asthma symptoms outcome as having ≥3 asthma symptoms. The two definitions were combined as having current asthma and/or ≥3 asthma symptoms. The respiratory outcomes of offspring were defined based on an affirmative answer to the respective questions (Supplementary Table S1).

Various epidemiological and clinical studies have suggested a close connection between asthma and allergic rhinitis (11–13). Allergic asthma was defined as current asthma and/or ≥3 asthma symptoms in addition to nasal allergy (defined as a positive response to “Do you have any nasal allergies including hay fever?”). Similarly, non-allergic asthma was defined as having asthma without a nasal allergy.

Personal TB was defined as a positive answer to the question, “Have you ever had tuberculosis?” which was included in the most recent follow-up of the RHINE study (RHINE IV). Parental smoking was defined based on the response to the questions “Did your father smoke regularly during childhood?” and “Did your mother ever smoke regularly during your childhood?”, included in the RHINE II questionnaire. Parental asthma was defined based on the questions in RHINE II: “Did your biological parents ever suffer from any of the following: Asthma (Mother/Father)”. Questions related to parental education was included in RHINE II: “What education did your mother have?” and “What education did your father have?”.

### 2.3. Statistical analyses

We used the currently available evidence and literatures to identify the minimal covariate adjustment set, and we found parental asthma, parental smoking habits, and socioeconomic status as potential confounders. Parental education was used as a proxy for socioeconomic status. In the supplementary tables, we included sensitivity analysis with adjustments for offspring's TB status.

Descriptive analyses were stratified by parental TB status. Unadjusted and adjusted logistic regression analyses were performed to study the association of parental TB with offspring respiratory outcomes. The odds ratio (OR) with a 95% confidence interval (CI) was calculated for both unadjusted and adjusted models. Analyses were performed separately for the paternal and maternal TB status, with further stratification based on the participant's sex for possible sex-specific inter-generational associations. All the statistical analyses were performed using Stata/SE 17.0 (Stata Corp, College Station, TX, USA).

## 3. Results

In total, 8,323 participants were included in the analyses. The median age of the participants was 62 years, ranging from 49 to 76 years; 53% were female. A total of 227 (2.7%) and 282 (3.4%) participants reported only paternal and maternal TB, respectively, while 33 (0.4%) participants reported that both parents had TB (Table 1). The participants' responses regarding the TB status of their parents, stratified by the participants' sex is presented in Supplementary Table S2 and the birth year of parents with TB is presented in Supplementary Table S3.

**Table 1:** Distribution and basic characteristics of participants according to the parental TB status.

	Father with TB (*n* = 227)	Mother with TB (*n* = 282)	Both with TB (*n* = 33)	Neither parent with TB (*n* = 7,781)	Total (*n* = 8,323)
Centre, *n*					
Aarhus	17	19	1	841	878
Bergen	30	31	3	1,411	1,475
Gothenburg	15	15	0	810	840
Reykjavik	51	82	11	1,269	1,413
Tartu	46	43	7	920	1,016
Umeå	27	33	6	999	1,065
Uppsala	41	59	5	1,531	1,636
Sex, *n* (%)					
Male	84 (37.0)	105 (37.2)	16 (48.5)	3,701 (47.6)	3,906 (46.9)
Female	143 (63.0)	177 (62.8)	17 (51.5)	4,080 (52.4)	4,417 (53.1)
Birthyear, range	1946–1973	1946–1973	1946–1973	1945–1973	1945–1973
Age (median, range)	63 (49–76)	64 (49–75)	63 (49–76)	62 (49–76)	62 (49–76)
BMI, mean (SD)	26.7 (5.7)	27.2 (5.1)	28.0 (4.9)	26.7 (4.6)	26.8 (4.7)
Respiratory outcomes, *n* (%)					
Have or ever had asthma	33 (14.5)	56 (19.8)	10 (30.3)	1,057 (13.6)	1,156 (13.9)
Attack of asthma^a^	18 (7.9)	16 (5.7)	4 (12.1)	335 (4.3)	373 (4.5)
Currently taking asthma medication^a^	34 (14.9)	37(13.1)	9 (27.3)	849 (10.9)	929 (11.2)
Current asthma	35 (15.3)	40 (14.1)	9 (27.3)	911 (11.7)	995 (11.9)
≥3 asthma symptoms score	39 (17.1)	56 (19.8)	7 (21.2)	1,144 (14.7)	1,246 (14.9)
Current asthma and/or ≥3 asthma symptoms	54 (23.7)	72 (25.4)	12 (36.4)	1,600 (20.5)	1,738(20.9)
Personal TB	10 (4.4)	5 (1.8)	3 (9.1)	37 (0.5)	55 (0.7)
Parental education (any of the parents), *n* (%)					
Primary school	61 (27.2)	97 (34.3)	10 (30.3)	2,815 (36.2)	2,983 (35.8)
Secondary school	86 (37.7)	93 (33.2)	9 (27.3)	2,210 (28.4)	2,398 (28.8)
College or university	50 (21.9)	57 (20.1)	8 (24.2)	1,505 (19.3)	1,620 (19.5)
Do not know of both	30 (13.2)	35 (12.4)	6 (18.2)	1,251 (16.1)	1,322 (15.9)
Parental smoking (at least one parent)	121 (53.3)	152 (53.9)	13 (39.4)	3,949 (50.7)	4,235 (50.9)
Parental asthma (at least one parent)	33 (14.5)	46 (16.3)	3 (9.1)	830 (10.7)	912 (10.9)

TB, tuberculosis; BMI, body-mass index; SD, standard deviation. Among female participants, 268 and 395 responded “Don't know” to maternal and paternal TB questions, respectively. While, among male participants, 449 and 374 responded “Don't know” to maternal and paternal TB questions, respectively.

^a^ In the last twelve months.

Overall, participants who reported parental TB had a higher prevalence of asthmatic outcomes and personal TB. Approximately 30% of participants who reported both parents with a history of TB also reported that they have or ever had asthma, compared to 13% of participants who reported that neither of their parents had TB. Furthermore, 4.4% of participants with paternal TB reported personal TB, while only 0.5% of participants without a history of parental TB reported personal TB (Table 1).

Current asthma (aOR: 1.34, 95% CI: 1.05–1.71), ≥3 asthma symptoms (aOR: 1.31, 95% CI: 1.05–1.65), and Current asthma and/or ≥3 asthma symptoms (aOR: 1.29, 95% CI: 1.05–1.57) were more common in participants with parental TB compared to neither parent having TB. Parental TB was significantly associated with allergic asthma in offspring, while no significant association between parental TB and asthma without allergy in offspring was observed (Table 2). Furthermore, statistically significant association was found between parental TB and wheezing among offspring (aOR: 1.42, 95% CI: 1.16–1.74).

**Table 2:** Asthma and respiratory symptoms as associated with parental TB.

	Crude		Adjusted^a^	
	OR (95% CI)	*P* value	OR (95% CI)	*P* value
Wheezing	1.44 (1.18–1.76)	<0.000	1.42 (1.16–1.74)	0.001
Awoken with tightness in chest	1.26 (0.97–1.62)	0.078	1.23 (0.95–1.59)	0.109
Awoken with attack of cough	1.27 (1.05–1.54)	0.013	1.25 (1.03–1.52)	0.021
Difficult breathing when walking on ground level	1.24 (1.01–1.52)	0.033	1.25 (1.02–1.53)	0.032
Shortness of breath when active	1.17 (0.98–1.39)	0.074	1.15 (0.97–1.37)	0.111
Current asthma	1.38 (1.08–1.76)	0.009	1.34 (1.05–1.71)	0.019
≥3 asthma symptoms	1.34 (1.07–1.68)	0.010	1.31 (1.05–1.65)	0.017
Current asthma and/or ≥3 asthma symptoms	1.32 (1.07–1.61)	0.007	1.29 (1.05–1.57)	0.015
Current asthma and/or ≥3 asthma symptoms and nasal allergy	1.64 (1.27–2.12)	<0.000	1.58 (1.29–2.05)	<0.000
Current asthma and/or ≥3 asthma symptoms and no nasal allergy	1.02 (0.77–1.33)	0.881	1.00 (0.76–1.32)	0.972

OR, odds ratio; CI, confidence interval.

^a^ Adjusted for parental education, parental smoking habits, parental asthma.

When stratifying by parent's sex, associations with offspring asthma were found for both maternal and paternal TB, there were no clear differences between the maternal and paternal lines although one could suspect more consistent results in the maternal line (Table 3). With stratification by offspring's sex, associations between parental TB and asthmatic conditions were statistically significant and indicated somewhat stronger estimates with regard to female as compared to male offspring, however, the confidence intervals were widely overlapping. Stratification by both parents' and offspring sex did not reveal clear sex-specific patterns, although associations appeared to be less consistent in the father-son line than in the three other groups (Table 3).

**Table 3:** Association of parental TB with offspring asthma, stratified by parent's sex, offspring sex, and both parental and offspring's sex.

	Current asthma		≥3 asthma symptoms		Current asthma and/or ≥3 asthma symptoms	
	aOR (95% CI)	*P* value	aOR (95% CI)	*P* value	aOR (95% CI)	*P* value
Parental lines						
Maternal	1.38 (1.01–1.90)	0.042	1.55 (1.17–2.07)	0.002	1.45 (1.12–1.88)	0.004
Paternal	1.57 (1.12–2.19)	0.009	1.31 (0.95–1.82)	0.103	1.36 (1.02–1.81)	0.038
Offspring's sex						
Female	1.34 (0.99–1.80)	0.056	1.35 (1.02–1.77)	0.033	1.33 (1.03–1.70)	0.026
Male	1.25 (0.81–1.94)	0.306	1.13 (0.75–1.71)	0.546	1.13 (0.79–1.61)	0.470
Parent-offspring sex						
Mother-daughter	1.36 (0.92–1.99)	0.119	1.55 (1.09–2.18)	0.014	1.43 (1.03–1.97)	0.030
Mother-son	1.34 (0.77–2.33)	0.301	1.42 (0.85–2.37)	0.182	1.40 (0.90–2.17)	0.130
Father-daughter	1.42 (0.93–2.16)	0.106	1.39 (0.94–2.05)	0.100	1.41 (0.99–2.01)	0.056
Father-son	1.85 (1.07–3.21)	0.028	1.09 (0.59–2.01)	0.780	1.21 (0.74–2.01)	0.437

Model adjusted for parental education, parental smoking habits, parental asthma. aOR, adjusted odds ratio; CI, confidence interval.

In a sensitivity analysis, we investigated whether the associations of parental TB with offspring asthma could be mediated by offspring TB (Supplementary Table S4). The findings were consistent with adjustment for personal TB status, suggesting direct effects of parental TB on offspring asthma, rather than mediation by personal TB.

## 4. Discussion

This is one of the first studies addressing the association of maternal and paternal TB with offspring asthma and respiratory symptoms. Our study found that parental TB was associated with higher odds of offspring asthma and respiratory symptoms compared to offspring with no parents with TB. These associations appeared to be similar for the paternal and maternal lines.

The literature on parental TB infection and its impact on offspring's respiratory health is very limited. The increased risk of asthma in offspring associated with parental TB in our study is supported by findings in a register-based study. López-Cervantes et al. investigated asthma and rhinitis in the offspring of parents with a history of TB, revealing that parental TB in childhood was associated with a higher risk of asthma in future offspring as compared to TB occurring after the birth of the offspring (10). The authors reported sex-specific patterns with stronger associations in the maternal line and the daughters. In the present study, sex-specific patterns were not clear, but a subtle indication of slightly stronger or more consistent estimates in the maternal/mother-daughter lines agree with the previous registry-based study.

Studies suggest that early life exposures, including exposure *in utero*, affect asthma and other allergic diseases (16, 17). Not only maternal line but paternal line exposures are also linked to asthma (18, 19). Growing evidence suggests that parental exposure to environmental factors *before conception* can modify the offspring's phenotype (20–22). Our findings support the general hypothesis that parental exposures *before conception* could influence future generations. Regarding parental infections and offspring outcomes, there are murine studies demonstrating effects of parental infection on offspring phenotype (7–9), for instance, through epigenetic mechanisms such as alterations in sperm small RNA profiles. In line with the murine studies, our study of humans indicates that TB-infection known to cause profound immunological changes—could possibly influence the next generation's phenotype. This is also supported by a human study by Jogi et al., in which parental *Toxocara* seropositivity was found to be associated with an increased risk of wheezing and allergies in offspring. The associations were stronger in the father-daughter and mother-son lines (23); which could possibly indicate sex-specific epigenetic mechanisms for transfer across generations. Regarding the present analysis of parental TB, we thus speculate that epigenetic modifications in germline cells due to the immunological impact of TB could be of importance for the observed effects in offspring (20).

There are studies suggesting an association of own TB infection with respiratory symptoms (24, 25). We performed a sensitivity analysis with the adjustment for personal TB status; the results were consistent and strong even after such adjustment. Undernutrition is an important risk factor for TB (26), which in sequence may impact personal respiratory status. However, we have not found any indication of this being relevant with regard to offspring's asthma. Therefore, the nutritional status of parents was not considered in the present study. It is generally accepted that parental asthma increases the risk of offspring asthma (27, 28). In the current study, the association between parental TB and offspring asthma was still significant after adjusting for parental asthma.

One strength of this study is the inclusion of participants from various Nordic-Baltic countries with different time trends in the prevalence of tuberculosis and asthma. Further, our analysis was based on a large population-based cohort study and using standardized procedures. Additionally, we characterized asthma based on the widely used ECRHS questions on asthma and respiratory symptoms. We used different definitions of asthma and respiratory symptoms: Doctor's diagnosed asthma may be more specific but may be influenced by the doctor's bias. Various studies have suggested that asthma defined based on asthma symptoms scores may be a very good measure to define asthma (14). We also combined “current asthma” and “asthma symptoms” based on such a score, into one variable (current asthma and/or ≥3 asthma symptoms) in order to increase the sensitivity. In general, our results were robust across different asthma definitions strengthening somewhat the conclusions.

The major limitation of this study is the use of a questionnaire where offspring reported parental TB. It is likely that some offspring may not bear in mind whether their parents had TB or not, leading to recall bias that may introduce misclassification. However, the misclassification is likely to be non-differential as we believe the participants' asthma or respiratory symptoms will probably not have influenced the recall of parents' TB status. Other respiratory diseases may be misdiagnosed as asthma due to partly overlapping symptoms and lack of more precise diagnostics. Therefore, some participants in our study who were classified as having asthma may actually have had other respiratory diseases. Since TB is very rare and asthma very common in our study population, such misclassification of asthma would be unlikely to alter the observed associations between TB and asthma. With regard to misclassification linked to common viral infections, such misclassification would tend to attenuate associations. As most of the parents were born in the early 1900s, and TB was endemic in the Nordic region at that time, it seems likely that most parents would have had the infection before the conception of the offspring; however, we cannot claim this with certainty. We did not have information on whether parental asthma preceded parental TB infection or not, limiting the possibility for more detailed analyses; however, the analyses were adjusted for parental asthma so this would not have biased the reported results.

## 5. Conclusion

Results from this study indicate that parental TB might be a risk factor for offspring′s asthma and respiratory symptoms. Based on our findings and previous murine studies, we speculate that the profound immunologic response to TB in parents might lead to epigenetic modification of germline cells and thereby influencing offspring phenotype. Our hypothesis-generating results should be investigated further, both in epidemiological and mechanistic studies.

## Data Availability

The datasets presented in this article are not readily available because of potential privacy violations, but they are made available upon justifiable request and with the consent of the relevant national ethics committee. Requests to access the datasets should be directed to https://rhine.w.uib.no/.
