# Supplementary material for: Maternal and paternal tuberculosis is associated with increased asthma and respiratory symptoms in their offspring: a study from Northern Europe
Source: Front Allergy. 2023 Jun 8;4:1193141. doi: 10.3389/falgy.2023.1193141 (PMC10286510; doi:10.3389/falgy.2023.1193141)
Supplement: Supplementary file 1 [file Table1.docx]

Supplementary Material

Maternal and paternal tuberculosis is associated with increased asthma and respiratory symptoms in their offspring: a study from Northern Europe

Sanjay Gyawali^*^, Juan Pablo López-Cervantes, Ane Johannessen, Thorarinn Gislason, Mathias Holm, Christer Janson, Rain Jögi, Lars Modig, Vivi Schlünssen, Tehmina Mustafa, Cecilie Svanes

# * Correspondence: [Sanjay.gyawali@gmail.com](https://universityofbergen-my.sharepoint.com/Users/sanjay/Downloads/Sanjay.gyawali@gmail.com)

[sgy002@uib.no](mailto:sgy002@uib.no)

Supplementary Tables

**Table S1: Respiratory symptoms and their definitions**

|  | Questions used to define respiratory symptoms |
| --- | --- |
| Wheezing | Have you had wheezing or whistling in your chest at any time in the last 12 months? |
| Wheezing with shortness of breath | Have you been at all breathless when the wheezing noise was present? |
| Wheezing without cold | Have you had this wheezing or whistling when you did not have a cold? |
| Awoken with tightness in chest | Have you woken up with a feeling of tightness in your chest at any time in the last 12 months? |
| Awoken with shortness of breath | Have you been woken by an attack of shortness of breath at any time in the last 12 months? |
| Awoken with an attack of cough | Have you been woken by an attack of coughing at any time in the last 12 months? |
| Shortness of breath when active | Do you get breathless with strenuous exercise? |
| Difficult breathing when walking on ground level | Do you get short of breath when hurrying on the level or walking up a slight hill? |
| Breathlessness | Do you walk slower than people of the same age on the level because of breathlessness, or do you have to stop for breath when walking on your own pace on the level? |
| Shortness of breath while walking | Do you stop for breath after walking about 100 metres or after a few minutes on the level? |
| Phlegm | Do you usually bring up phlegm or do you have phlegm in your lungs which you have difficulty bringing up? |
| Nasal allergies | Do you have any nasal allergies including hay fever? |
| Attack of asthma | Have you had an attack of asthma in the last 12 months? |
| Currently taking asthma medication | Are you currently taking any medicine (including inhalers, aerosols, or tablets) for asthma? |

**Table S2: Participants' response to whether their parents were ever treated for tuberculosis stratified by sex.**

|  | Mother | | | Father | | | Both  Yes |
| --- | --- | --- | --- | --- | --- | --- | --- |
|  | Yes | No | Do not know | Yes | No | Do not know |  |
| Male | 121 | 3 394 | 375 | 100 | 3 285 | 449 | 16 |
| Female | 194 | 3 933 | 268 | 160 | 3 798 | 395 | 17 |

**Table S3: Birth year of parents with TB**

| Year | Mother (n) | Father (n) |
| --- | --- | --- |
| Before 1910 | 4 | 15 |
| 1910-1919 | 41 | 46 |
| 1920-1929 | 114 | 96 |
| 1930-1939 | 100 | 62 |
| After 1939 | 44 | 29 |
| Do not know | 12 | 12 |
| Total | 315 | 260 |

**Table S4: Association between parental tuberculosis and asthma, adjusted by personal TB status.**

|  | Crude | | Adjusted* | |
| --- | --- | --- | --- | --- |
|  | OR (95% CI) | P Value | OR (95% CI) | P Value |
| Current asthma | 1.38 (1.08-1.76) | 0.009 | 1.35 (1.05-1.73) | 0.016 |
| ≥ 3 asthma symptoms | 1.34 (1.07-1.68) | 0.010 | 1.29 (1.02-1.62) | 0.030 |
| Current asthma and/or ≥ 3 asthma symptoms | 1.32 (1.07-1.61) | 0.007 | 1.28 (1.04-1.57) | 0.017 |

*Adjusted for personal TB status

OR: Odds ratio CI: confidence interval
